# Alterations to Dendritic Spine Morphology, but Not Dendrite Patterning, of Cortical Projection Neurons in Tc1 and Ts1Rhr Mouse Models of Down Syndrome

**DOI:** 10.1371/journal.pone.0078561

**Published:** 2013-10-30

**Authors:** Matilda A. Haas, Donald Bell, Amy Slender, Eva Lana-Elola, Sheona Watson-Scales, Elizabeth M. C. Fisher, Victor L. J. Tybulewicz, François Guillemot

**Affiliations:** 1 Division of Molecular Neurobiology, Medical Research Council National Institute for Medical Research, London, United Kingdom; 2 Confocal Image Analysis Laboratory, Medical Research Council National Institute for Medical Research, London, United Kingdom; 3 Division of Immune Cell Biology, Medical Research Council National Institute for Medical Research, London, United Kingdom; 4 University College London Institute of Neurology, London, United Kingdom; Universidade Federal do ABC, Brazil

## Abstract

Down Syndrome (DS) is a highly prevalent developmental disorder, affecting 1/700 births. Intellectual disability, which affects learning and memory, is present in all cases and is reflected by below average IQ. We sought to determine whether defective morphology and connectivity in neurons of the cerebral cortex may underlie the cognitive deficits that have been described in two mouse models of DS, the Tc1 and Ts1Rhr mouse lines. We utilised in utero electroporation to label a cohort of future upper layer projection neurons in the cerebral cortex of developing mouse embryos with GFP, and then examined neuronal positioning and morphology in early adulthood, which revealed no alterations in cortical layer position or morphology in either Tc1 or Ts1Rhr mouse cortex. The number of dendrites, as well as dendrite length and branching was normal in both DS models, compared with wildtype controls. The sites of projection neuron synaptic inputs, dendritic spines, were analysed in Tc1 and Ts1Rhr cortex at three weeks and three months after birth, and significant changes in spine morphology were observed in both mouse lines. Ts1Rhr mice had significantly fewer thin spines at three weeks of age. At three months of age Tc1 mice had significantly fewer mushroom spines - the morphology associated with established synaptic inputs and learning and memory. The decrease in mushroom spines was accompanied by a significant increase in the number of stubby spines. This data suggests that dendritic spine abnormalities may be a more important contributor to cognitive deficits in DS models, rather than overall neuronal architecture defects.

## Introduction

Down Syndrome (DS) is a complex human genetic disorder, caused by to the presence of a third copy of up to 300 genes from an extra human chromosome 21 (Hsa21). While a spectrum of clinical phenotypes can result, one feature consistent in all DS is the intellectual deficit that impairs learning and memory. Similar deficits have been demonstrated in DS mouse models. Defects in embryonic and postnatal cerebral cortex development are likely to underlie these deficits, but are yet to be fully identified.

**Figure 1:**
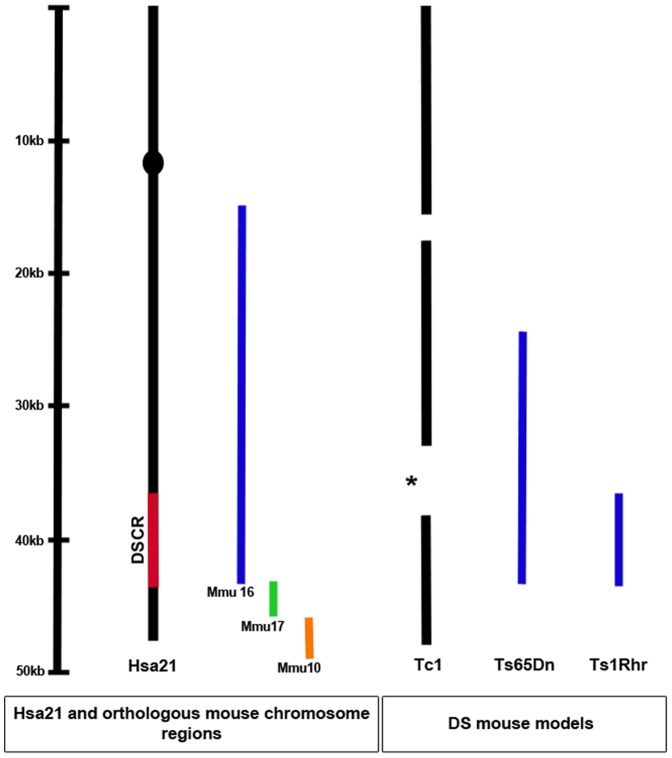
Hsa21, orthologous mouse chromosome regions and relevant DS mouse models. The proposed Down Syndrome Critical Region (DSCR), a region of approximately 33 genes formerly thought to be sufficient to produce DS phenotypes, is located on the long arm of Hsa21. Hsa21 is syntenic with regions of mouse chromosomes 16, 17 and 10. The Tc1 mouse carries a freely segregating copy of Hsa21, but suffers from regions of deletion (the two largest are shown) and duplication. The asterisk represents the deleted region where genes involved in synaptic development (*ITSN, SYNJ* and *DSCR1*) are located. Ts65Dn is a duplication of approximately 140 Mmu16 genes. Ts1Rhr is a duplication of the Mmu16 region corresponding to the DSCR.

Abnormal dendritic arborisation would impact on a neuron's participation in the cortical circuitry and potentially contribute to learning and memory deficits in DS and DS mouse models. Studies using human tissue indicate that dendritic arborisation is affected in DS, but in a case specific manner [1]. Mouse models have been more demonstrative. Cortical basal dendrites in the Ts65Dn mouse brain are shorter and less branched than wildtypes [2]. *DYRK1A*, a gene localised to the so-called Hsa21 Down Syndrome Critical Region (DSCR; Figure 1), which is present in three copies in Ts65Dn mice, is a candidate gene for dendritic branching defects [3], [4]. *DYRK1A* encodes a protein kinase of the Dual-specificity tyrosine phosphorylation-regulated kinase family, and regulates several signalling pathways in brain development, from progenitor proliferation through to terminal differentiation (reviewed by [5]).

Dendritic spines, the sites of projection neuron excitatory synaptic inputs, may also be affected in DS [6], [7]. One study inferred from human DS cases that spines increase in number normally, but then rapidly decrease after 20 years of age [8]. Unusually enlarged spine heads have been observed in motor cortex of DS mouse models [2], [9], [10], [11] as well as decreased spine density in the hippocampal dentate gyrus [9], [10].

Genetically engineered mouse models have advanced our ability to investigate mechanisms of DS. Models of near complete trisomy include Tc(Hsa21)1TybEmcf, which is a transchromosomic mouse strain that contains Hsa21 (hereafter Tc1) [12], Ts1Yey, Ts2Yey and Ts3Yey strains carry duplications of the regions of mouse chromosome 16 (Mmu16), Mmu17 and Mmu10 orthologous to Hsa21 [13], [14], while models trisomic for shorter segments of the mouse chromosomes orthologous to Hsa21, include the widely-used Ts65Dn and Ts1Rhr strains [15], [16]. Learning and memory deficits have been confirmed in Tc1, Ts65Dn, Ts1Rhr and Ts1Yey mice [10], [12], [14],[17],[18].

We sought to determine whether abnormal morphology of cortical neurons underlies learning and memory deficits in DS mouse lines, and to compare the contribution of the DSCR alone (Ts1Rhr), or most of Hsa21 (Tc1), to observed phenotypes. We identified no significant difference in the length or branching of neuronal dendrites in Layer II-IV cortical projection neurons from Tc1 or Ts1Rhr mouse brains. However, projection neurons within the cortices of both Tc1 and Ts1Rhr mice showed significant alterations in spine morphology. Our data reports on the novel finding that alterations to dendritic spine morphology may be a significant contributor to phenotypes in these DS models rather than changes in overall dendritic architecture.

## Materials and Methods

### Animals

This study was conducted following approval by the local Ethical Review Process of the MRC National Institute for Medical Research and authorisation by the UK Home Office, Animals (Scientific Procedures) Act 1986 under relevant Project License authority. The ERP approved the work and reported that all work reflects contemporary best practice. Tc1 mice were maintained on a 129S8:C57BL/6J (F1) background [12]. For experiments 129S8:C57BL/6J (F1) Tc1 female mice were time-mated with C57BL/6J males, and 129S8:C57BL/6J (F1) Ts1Rhr males were time-mated to C57BL/6J female mice. The result of these mating schemes was that all mice were effectively backcrossed twice to C57BL/6J. In all cases control wild-type mice were littermates of mutant mice analysed. Genotyping was performed by Polymerase Chain Reaction (PCR) for all strains. Tc1 genotyping has been previously described [12]. Ts1Rhr genotyping was determined by PCR analysis using primers CCGTCAGGACATTGTTGGA and CCGTAACCTCTGCCGTTCA (Reeves, unpublished).

### Expression constructs

pCALNL-GFP, pCALNL ERT-Cre-ERT and pCAG-RFP plasmid DNA expression constructs were obtained from the Cepko lab [19]. The details can be obtained from http://www.addgene.org/. A GFP expression construct under the control of a Tamoxifen inducible Cre was specifically chosen to take advantage of the use of low doses of Tamoxifen to limit the number of GFP expressing cells, thus facilitating more precise imaging and analysis of individual neurons. pCAG-RFP expression was used to screen successfully electroporated cortices, because GFP expression was not induced by tamoxifen activation of ERT-Cre-ERT until post-natal stages.

### In utero electroporation and tissue preparation

*In utero* electroporation was performed based on methodology developed by Tabata and Nakajima [20] that we have previously described [21], [22]. We used a Cre inducible GFP expression vector, combined with a low dose tamoxifen, to limit the number of GFP expressing neurons, which facilitated more accurate analysis of the neuronal morphology (dendritic arbor and dendritic spines). Briefly, *in utero* electroporation surgery was performed at E15, and the embryos were returned to the mother and their development allowed to progress normally. Parkes foster mothers were routinely used to increase the survival of neonates born from mutant mouse strains following the surgery. A low dose of Tamoxifen (10 µg/g body weight; Sigma) was administered intraperitoneally to mice at P7. At P21 or 3 months of age mice were deeply anaesthetised with sodium pentobarbitone (800 µg/10 g body weight) and transcardially perfused with 5–10 mL phosphate buffered saline (PBS) followed by 20–30 mL 4% paraformaldehyde (PFA). Brains were removed and post-fixed overnight in 4% PFA. Brains were embedded in Agarose and then coronally sectioned at 100 µm thickness using a vibratome (Leica). Sections were mounted to slides, dried and coverslip mounted with Aquapolymount (Polysciences). Imaging of neuronal architecture and dendritic spines was routinely performed using native GFP fluorescence.

### Immunohistochemistry

Free floating 100 µm sections were blocked and permeabilised (PBS/10% Normal Donkey Serum/0.1% Triton) for one hour prior to the addition of primary antibodies. Anti-mouse NeuN (1∶500; Chemicon) and anti-sheep GFP (1∶100; MorphoSys (Biogenesis)) were applied and incubated for 2 days at room temperature. Following extensive washing (PBS/0.01% Triton), sections were incubated for two hours with the appropriate Alexa Fluor fluorescent secondary antibodies (1∶500; Molecular Probes). Sections were washed with PBS and mounted using Aquapolymount.

### Microscopy and Analysis

GFP positive Layer II-IV projection neurons were imaged from motor 1 and somatosensory 1 areas, restricted to rostro-caudal axis co-ordinates Bregma 1.10 mm to −0.10 mm, using a limited range of gain and offset settings and a Leica SP5 confocal microscope equipped with a 20/0.7NA objective. High resolution imaging of dendritic spines was performed using a 100/1.46NA oil immersion objective combined with 4× zoom. Approximate 40 µm length basal dendrite segments were imaged at an initial distance of 75 µm from the cell body. Images were reconstructed for analysis using a combination of ImageJ (1.44) and Volocity software (6.0). Neuron morphology was reconstructed by tracing through the depth of the Z-stack using Neurolucida (8.21.8). Neuron morphology was analysed in part by Sholl analysis, in which concentric circles of increasing distance (in this case 20 µm) from the cell body are aligned over the image of the neuron and the number of times the dendrites intersect circles at each radii is calculated (as a measure of the degree of branching), as well as the total dendrite length lying within each circle [23]. Dendritic spine density, size and morphological classifications were obtained using Neuron Studio software [24]. Statistical analysis was performed using GraphPad Prism (6.0c). Unpaired t-test was used for analysis when data passed the D'Agostino and Pearson normality test, otherwise the non-parametric Mann Whitney test was used. 2-way ANOVA with Bonferroni multiple comparisons post-test was used to compare Sholl analysis and dendritic spine classification data.

## Results

### Positioning of GFP labelled neurons is normal in Tc1 and Ts1Rhr Down syndrome models

**Figure 2:**
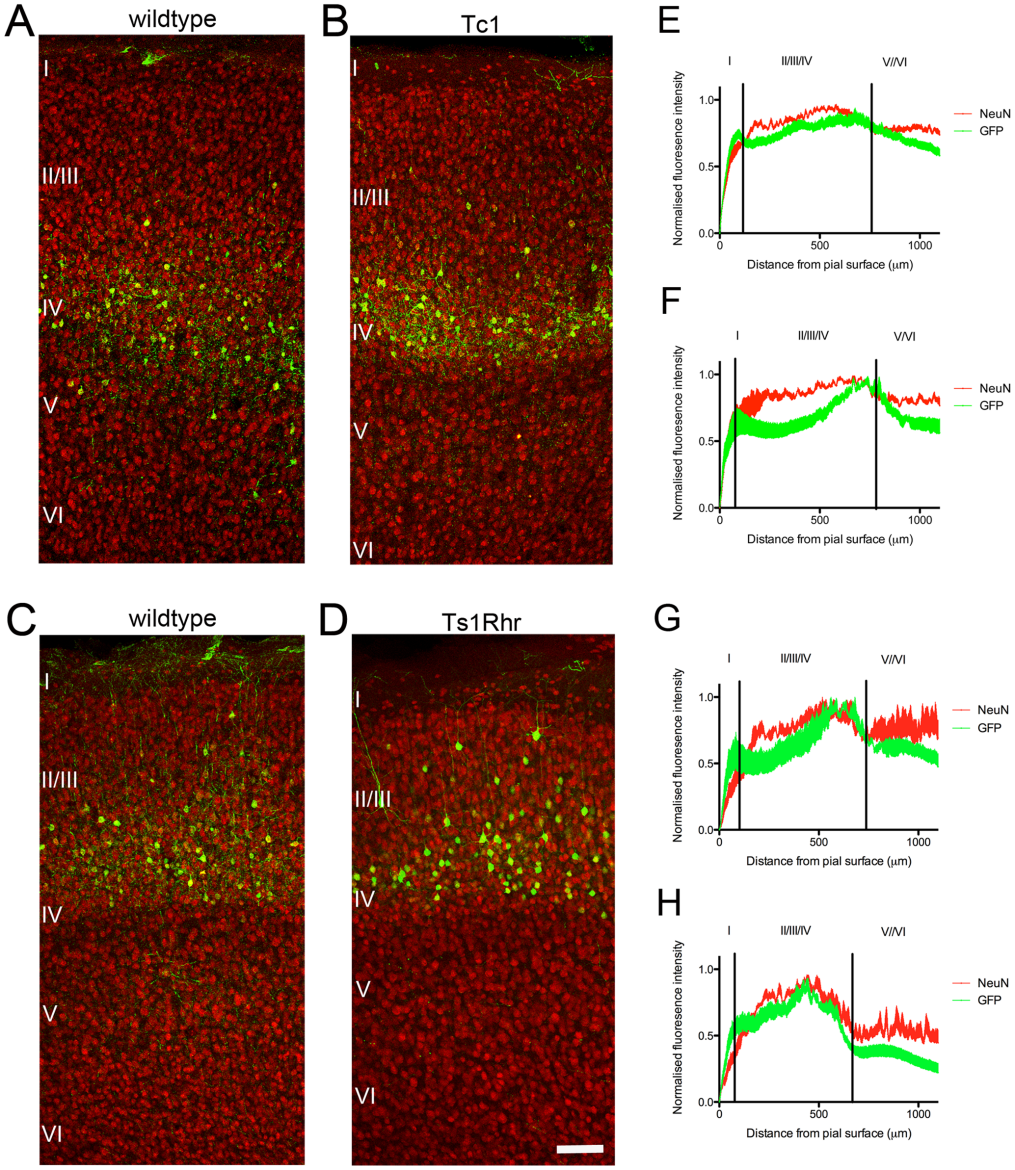
The distribution of GFP-electroporated neurons in Tc1 and Ts1Rhr mouse cortex. The cortex of E15 mice was electroporated with a GFP expression construct, and mice allowed to develop normally until P21, when the brains were harvested and immunolabelled with antibodies to GFP (green) and NeuN (red). ***A***. wildtype (Tc1) cortex ***B***. Tc1 cortex ***C***. wildtype (Ts1Rhr) cortex ***D***. Ts1Rhr cortex ***E-H***. Normalised fluorescence intensity profiles for GFP and NeuN labelling through the cortical layers. ***E***. wildtype (Tc1) cortex shows the peak of GFP labelling corresponding to layers II–IV ***F***. Tc1 GFP fluorescence was also highest through layers II–IV ***G***. wildtype (Ts1Rhr) cortex fluorescence intensity peaked in layers II–IV ***H***. Ts1Rhr cortex also shows peak expression in Layers II–IV. n = 3 animals per genotype. Scale bar  = 100 µm

Previous reports indicated that cortical lamination might be affected in human DS patients [25]. We labelled a cohort of early post-mitotic neurons at E15, by in utero electroporation with a GFP expression construct, to investigate potential abnormalities in cortical lamination in our DS mouse models. Coronal sections of GFP-electroporated brains from P21 mice were immunolabeled for GFP and NeuN, a mature neuronal marker, to assist in the histological identification of cortical layers (Figure 2A-D). In Tc1 brains (Figure 2B), the GFP fluorescence intensity profile (Figure 2E) showed E15 generated neurons were distributed similarly to wildtype controls (Figure 2A and F). The high density of NeuN in Layers II-IV corresponded to the region where most GFP positive neurons were located, as demonstrated in fluorescence intensity profiles ([21] Figure 2E, F). The distribution of GFP positive neurons in Ts1Rhr cortex (Figure 2D, H) was similar to wildtype controls (Ts1Rhr; Figure 2C, G). We noted a difference in the distribution of GFP expressing neurons between the Tc1 and Ts1Rhr mouse strains, potentially due to slight differences in the genetic background of the two strains. Whilst the fluorescent intensity appears to be decreased in the Ts1Rhr intensity profile, which could be for technical reasons, the overall cell distribution is the same, as indicated in the images.

Our data is consistent with previous reports showing that upper layer (II–III) mouse cortical projection neurons are generated between E15–E17, with a peak at E16 [26]. Thus our GFP positive neurons electroporated at E15 most likely represent the last layer IV neurons and some of the first generated Layer II/III neurons. Therefore, layering of upper cortical projection neurons in not affected in Ts1Rhr or Tc1 mice.

### Dendrite morphology in Layer II–IV projection neurons is not affected in Tc1 or Ts1Rhr mouse brains

**Figure 3:**
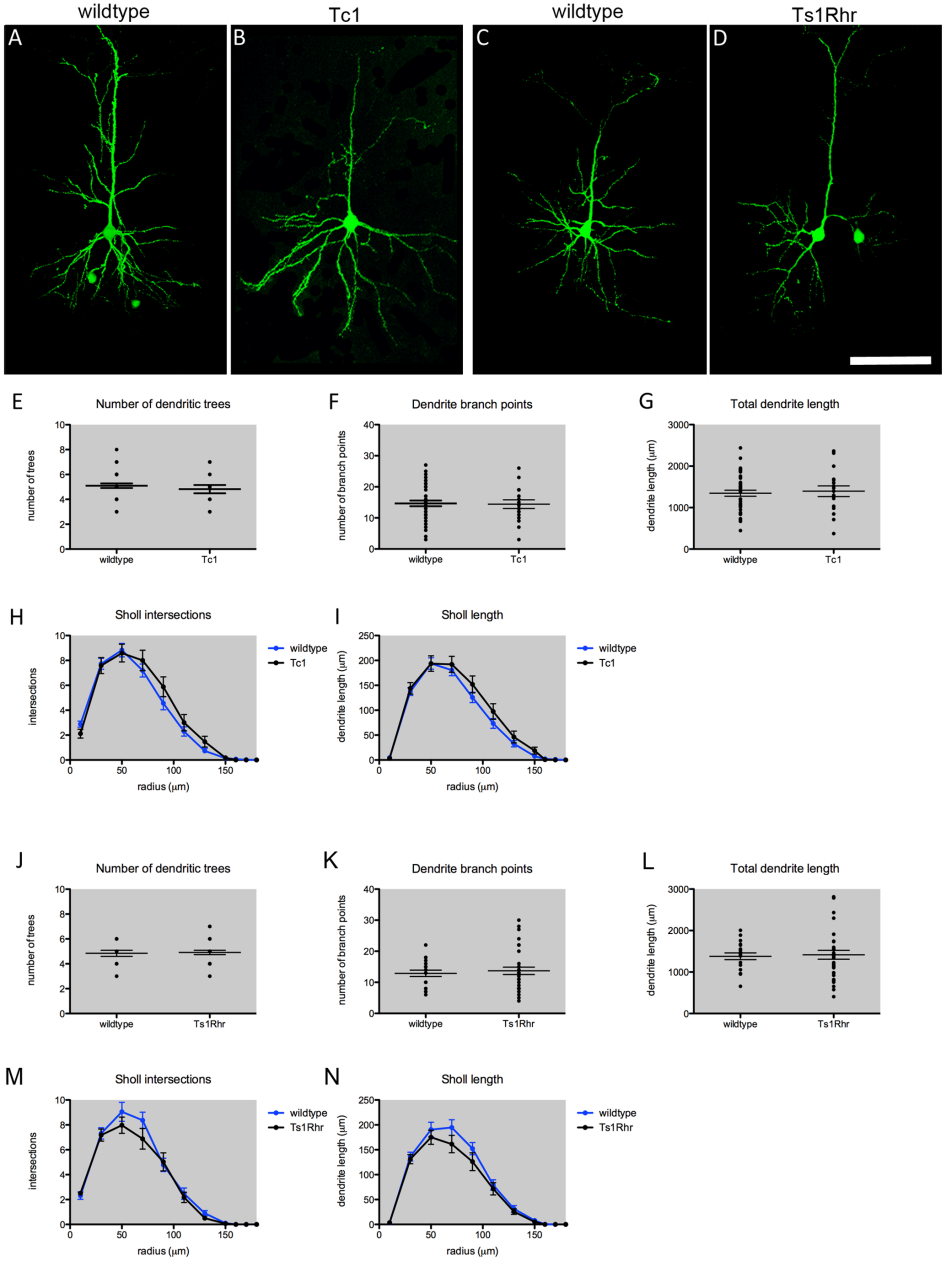
Layer II–IV cortical projection neuron morphology in Tc1 and Ts1Rhr mice. Brains from E15 GFP electroporated mice were harvested at P21. Thick coronal sections were imaged and the neuronal structure, particularly the dendritic arbor, was reconstructed to identify changes in neuronal morphology. ***A–D***. Representative images of Layer II–IV GFP+ projection neurons demonstrate heterogeneity in morphology within the projection neuron population. ***A***. wildtype (Tc1) ***B***. Tc1 ***C***. wildtype (Ts1Rhr) and ***D***. Ts1Rhr ***E*** **–** ***I*** **.** Analysis of the dendritic arbor in wildtype and Tc1 mouse cortex (n = ≥18 neurons from ≥4 mice per genotype). ***E***. The number of dendritic trees (primary branches) emanating from the cell body ***F***. Dendrite length ***G***. Number of branch points ***H***. Sholl analysis showing the number of intersections, a measure if branching complexity, with increasing distance from the soma. ***I***. The dendrite length with increasing distance from the soma. ***J*** **–** ***N***. Analysis of wildtype and Ts1Rhr dendrites (n = ≥19 neurons from ≥4 mice per genotype). ***J***. The number of dendritic trees ***K***. Dendrite length ***L***. Dendrite branching ***M***. Sholl analysis showing the number of intersections with increasing distance from the cell body ***N***. Sholl analysis showing dendrite length with increasing distance from the cell body. Scale bar  = 50 µm

Next we investigated whether the morphology of Layer II–IV projection neurons was affected in DS mouse models. GFP fluorescence was used to trace the *in vivo* structure of the entire neuron, including the apical and basal dendritic arbors. Neurons were traced from reconstructed confocal microscopy images taken of cells within 100 µm-thick brain slices, to ensure the 3D structure of neurons was preserved. We initially observed no striking differences in either polarity or orientation of the neurons within the cerebral cortex, in either Tc1 (Figure 3B), or Ts1Rhr (Figure 3D), compared with their respective controls (Figure 3A and C). Interestingly, a high degree of heterogeneity in terms of cell morphology was observed in the projection neuron population, as demonstrated in Figure 3, and this was also observed in all genotypes.

Analysis showed the total number of dendritic trees emanating from the cell body was not significantly different for Tc1 neurons compared to control (Figure 3E), or Ts1Rhr neurons compared to controls (Figure 3J). In addition, the total dendritic length and number of branch points was not affected in Tc1 (Figure 3F, G) or Ts1Rhr neurons (Figure 3K, L). Sholl analyses were carried out in order to identify more specific changes to branching with increasing distance from the cell body (as measured by the number of intersections and dendrite length). These analyses further demonstrated that the morphology of Tc1 cortical neurons to be very similar to control (Figure 3H, I), and Ts1Rhr neurons did not show any significant differences compared with wildtype control neurons (Figure 3M, N).

### Dendritic spine morphology is affected in Tc1 and Ts1Rhr mice

The density [9], [10] and morphology [2], [10], [11], [27] of dendritic spines have been reported to be abnormal in the hippocampus and cerebral cortex in DS mouse models.

**Figure 4:**
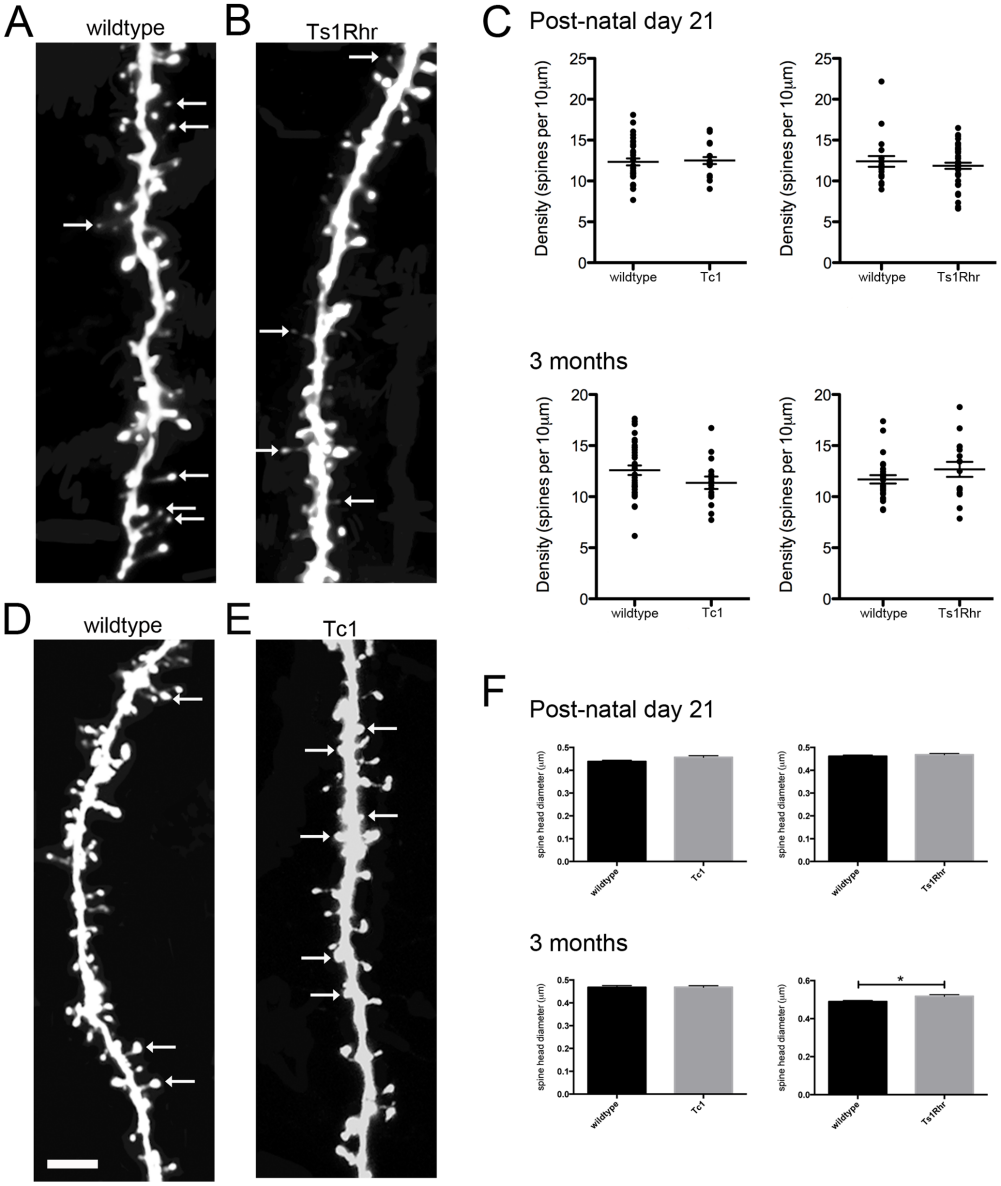
Dendritic spines in Tc1 and Ts1Rhr mouse cortex. GFP electroporated embryos at E15 were allowed to develop normally until P21 or 3 months of age. ***A***. Representative image of a wildtype (Ts1Rhr) dendrite segment, at P21. Arrows indicate thin spines. ***B***. Dendritic spines in Ts1Rhr cortex at P21. The representative image shows fewer thin spines compared with wildtype. ***C***. The dendritic spine density in Tc1 and Ts1Rhr cortex at P21 (≥19 neurons from ≥4 mice per genotype) and in 3-month-old mice (≥15 neurons from ≥3 animals per genotype). ***D***. A dendritic spine segment from wildtype (Tc1) cortex from a brain at 3 months of age. Arrows indicate spines of mushroom morphology. ***E***. A section of dendrite from the Tc1 cortex, with arrows indicating an increased number of stubby spines compared with wildtype controls. ***F***. Dendritic spine head diameter at P21 and 3 months, showing a significant increase in spine head diameter in Ts1Rhr cortex, compared to wildtype control (Ts1Rhr 0.5177 µm versus wildtype 0.4895 µm, p<0.05, Mann-Whitney test; n = >760 spines per genotype). *p<0.05. Scale bar  = 3.6 µm

We used GFP fluorescence expression to analyse the density and morphology of spines, in young adolescent (P21) and adult (3 month old) Tc1 and Ts1Rhr motor and somatosensory cortex. Short basal dendrite segments were imaged at high magnification for each genotype and we found no alterations in spine density in either Tc1 or Ts1Rhr mice at P21 or at 3 months of age (Figure 4C). The average spine head size was not significantly different in either DS mouse model at either P21 or 3 months (Figure 4F), but spine head diameter was significantly increased in neurons from the cortex of 3 month-old Ts1Rhr animals (Figure 4F). Previously, Belichenko and colleagues [27] also reported significantly enlarged spine heads in neurons of the motor cortex of Ts1Rhr mice.

**Figure 5:**
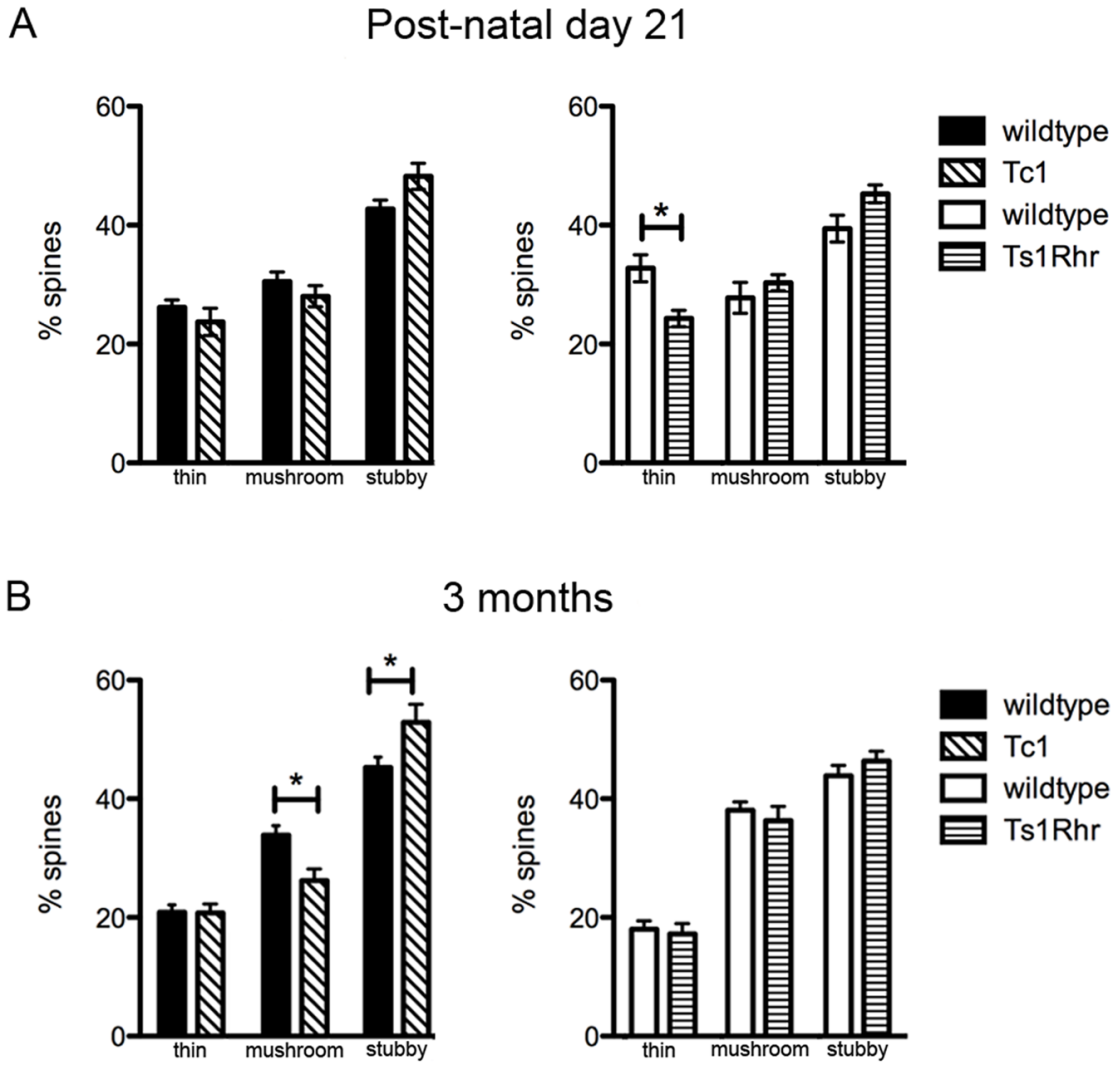
Dendritic spine classification by morphology, in Tc1 and Ts1Rhr mouse cortex. ***A***. At P21, there were significantly fewer thin spines in Ts1Rhr cortex at P21 (Ts1Rhr 24.30%±1.38, wildtype 32.77%±2.27; p<0.05; n = ≥19 neurons from ≥5 animals per genotype) ***B***. Dendritic spine classifications in 3-month-old mouse cortex shows significantly fewer mushroom spines (Tc1 26.25%±1.92, wildtype 33.86%±1.62; p<0.05; n = ≥15 neurons from ≥3 animals per genotype) but significantly more stubby spines in Tc1 cortex, compared with wildtype controls (Tc1 52.86%±3.07, wildtype 45.29%±1.74; p<0.05). *p<0.05.

Next we classified dendritic spines into thin, mushroom and stubby morphological categories. Spine shape is an indicator of the strength of synaptic input (eg reviewed by [28]), with mushroom spines thought to represent long-lasting synaptic inputs associated with learning and memory. We found at P21 there were significantly fewer thin spines in Ts1Rhr neurons, compared with control (Figure 4A, B and 5A). In contrast, no changes in spine morphology were observed in Tc1 mice at P21. By 3 months of age, Ts1Rhr spine proportions were within the range similar to wildtype (Figure 5B). In contrast, at 3 months of age, Tc1 neurons had significantly fewer mushroom spines with a concomitant increase in the number of stubby shape spines (Figure 4D, E and 5B). Therefore, our results indicate that while the number of dendritic spines is not different in Tc1 and Ts1Rhr mice compared with control, the dendritic spine morphology of Ts1Rhr and Tc1 neurons displays features consistent with defective synaptogenesis.

## Discussion

We wanted to determine whether changes to the morphology of cortical projection neurons, or their dendritic spines, underlie the learning and memory deficits in two mouse models of DS. We found no alterations in the dendritic outgrowth and branching patterns in Tc1 (which contain approximately 200 Hsa21 genes) or Ts1Rhr (duplication of 33 Mmu16 genes) mouse brains. Dendritic outgrowth and branching defects have been observed in cortical neurons in another DS mouse model, the Ts65Dn [2], in which approximately 140 Mmu16 genes are overexpressed [15]. One study previous to ours did examine dendritic morphology in Ts1Rhr [27], where a slight but significant decrease in thickness of the apical dendrite was observed, but no other changes to dendritic branching or outgrowth. Defects in Ts65Dn but not Ts1Rhr could indicate overexpression of gene(s) outside the DSCR is responsible, and why the defects would be observed in Ts65Dn but not Tc1 could potentially be due to a break in the human chromosome in Tc1, where a deletion from q32.56–35.29 occurred (asterisk, Figure 1). Trisomy of an additional 60 Mmu17 genes has recently been identified in the Ts65Dn mouse line [29] and so those genes must now be considered in any phenotypes associated with Ts65Dn.

A further possibility is that differences between mouse and human gene expression and function could introduce phenotypes not associated with the human disorder, and this demonstrates the value of the Tc1 model, the only DS model that expresses genes from the human chromosome. However, it should be noted that the Hsa21 in Tc1 mice has several regions that are duplicated or deleted, as well as a number of rearrangements [30]. These are thought to have resulted from irradiation damage that occurred during generation of the model. Nonetheless the Hsa21 in Tc1 mice carries 200 RefSeq genes in one copy and thus Tc1 mice have a total of three copies of these genes (one human and two mouse). As a further complication, Tc1 mice are mosaic, with around 50% of cells carrying the Hsa21 [12].

One further confounding difficulty in interpreting phenotypes from DS models is that phenotypes are dependent on the genetic background (eg [31]). The genetic background of Ts1Rhr mice in the study of Belichenko (B6EiC3Sn/J F1) [27] differed to the current study (129S8:C57BL/6J F1), the consequence of which may be changes in the severity or absence of phenotypes altogether.

The data available for human cases is difficult to interpret since the dendrite structure may actually be normal [32], and more complex or normal arborisation has been observed in neonates up to two years of age with DS [33]. It appears that in the years after birth and into adulthood, the dendrites are decreased in complexity compared with euploid cases [1], [34], suggesting a degenerative phenotype. As further demonstration of the complexity of the phenotype, Takashima (et al., [1]) presented two different age matched infant DS cases, one of which showed markedly increased, and the other drastically decreased dendritic arborisation compared with controls. There has been limited follow up for these observations, potentially because human tissue is scarce and matching controls for age and other clinical phenotypes is difficult for DS.

The mechanisms of DS remain largely unknown and correlating genotype to phenotype has largely relied upon what has been learned from gain or loss of function mutants for individual Hsa21 genes. Other Hsa21 candidate genes for controlling dendrite patterning are numerous and include *DSCAM* [35], [36], *TIAM1* [37], *APP* [38], [39], and *TTC3* [40].

Dendritic spine density is decreased in projection neurons of the hippocampus in human DS cases [41], as well as in DS mouse models (Ts65Dn and Ts1Rhr) [11], [27]. Defects in the DS mouse motor and somatosensory areas of the neocortex are more subtle, with no change in spine density, but changes in spine morphology observed – specifically an increase in the dendritic spine head size. We analysed mice at earlier time points than previous studies (3 weeks and 3 months), and observed increased spine head size at 3 months, but not 3 weeks of age. Indeed, it has been suggested that spine abnormalities in human DS are degenerative, rather than a developmental defect [6], and our current work therefore supports the idea that differences in spine characteristics emerge at later time points.

We classified spines using volumetric analysis as having thin, stubby or mushroom morphologies [24]. To our knowledge this is the first time such analysis has been carried out in a DS model. We were able to identify significant differences in both Ts1Rhr and Tc1 mice, compared to their respective controls. Most notably, an increase in stubby spines in Tc1 mice at three months of age, at the expense of mushroom spines, was observed, in contrast to a normal decrease in stubby spines and increase in mushroom spines with increasing age [42]. The Tc1 Hsa21 has been affected by several regions of deletion, and interestingly, one of those regions lost in Tc1 (and therefore not expressing a third copy of those genes) encompasses three genes previously implicated in synaptic development; *ITSN, SYNJ1* and *DSCR1* (Figure 1)[43]. This indicates that there are likely to be other key genes controlling spine morphogenesis and synaptic development on Hsa21. Further investigation is warranted to determine whether defective cytoskeletal or synaptic signalling mechanisms underlie spine morphology and behavioural deficits. For example, we note that the Rho GEF Tiam1, which is duplicated in Tc1 but not Ts1Rhr mice, has been reported to be involved in spine development and thus its increased dosage in Tc1 mice may contribute to the observed defects [37].

The difference in experimental approaches taken in previous studies, compared with ours, could influence the data and its interpretation. Previous approaches include the selective injection of Lucifer Yellow tracer into individual cells, which are filled with the dye through application of a current. Alternatively, Golgi-Cox impregnation has been used, which is not cell type specific, labelling neurons and glia in a random fashion. In contrast, we labelled a specific population of projection neurons generated at the dorsal ventricular zone of the telencephalon at E15 with GFP, which eventually populated cortical layers II–IV. Perhaps in restricting our analysis to such a specific population we have been unable to uncover the defects observed in a study of more heterogeneous populations. Because of these differences in the neurons being labelled, it is difficult, at present, to draw direct comparisons regarding the extent of filling of neuronal structures between the dye and GFP expression methods. However, we note that in our studies we found that the thickness of GFP-labelled apical oblique dendrite segments in 3 week-old wildtype mice was 3.21±0.03 µm (data not shown). In contrast, using Lucifer Yellow tracing, Belichenko et al reported that apical oblique dendrite thickness in 6 month-old mice was 0.80±0.02 µm [27]. This difference suggests that either the two labelling methods differ considerably, or that we have recorded the morphology of a different subset of neurons compared to the previous work, or that the difference was due to different ages of mice analysed.

In summary, our data supports the idea that defects in dendritic spine morphology may be an important contributor to DS phenotypes. In future studies it would be interesting to combine experimental learning and memory paradigms with analysis of dendritic spine plasticity, to determine whether the change in distribution of specific spine types is a cause or consequence of learning and memory deficits in these DS models.
